# A biochemical network modeling of a whole-cell

**DOI:** 10.1038/s41598-020-70145-4

**Published:** 2020-08-06

**Authors:** Paulo E. P. Burke, Claudia B. de L. Campos, Luciano da F. Costa, Marcos G. Quiles

**Affiliations:** 1 University of São Paulo, Bioinformatics Graduate Program, São Carlos, SP Brazil; 2 Institute of Science and Technology, Federal University of São Paulo, São José dos Campos, SP Brazil; 3 São Carlos Institute of Physics, University of São Paulo, São Carlos, SP Brazil

**Keywords:** Biochemical reaction networks, Data integration

## Abstract

All cellular processes can be ultimately understood in terms of respective fundamental biochemical interactions between molecules, which can be modeled as networks. Very often, these molecules are shared by more than one process, therefore interconnecting them. Despite this effect, cellular processes are usually described by separate networks with heterogeneous levels of detail, such as metabolic, protein–protein interaction, and transcription regulation networks. Aiming at obtaining a unified representation of cellular processes, we describe in this work an integrative framework that draws concepts from rule-based modeling. In order to probe the capabilities of the framework, we used an organism-specific database and genomic information to model the whole-cell biochemical network of the *Mycoplasma genitalium* organism. This modeling accounted for 15 cellular processes and resulted in a single component network, indicating that all processes are somehow interconnected. The topological analysis of the network showed structural consistency with biological networks in the literature. In order to validate the network, we estimated gene essentiality by simulating gene deletions and compared the results with experimental data available in the literature. We could classify 212 genes as essential, being 95% of them consistent with experimental results. Although we adopted a relatively simple organism as a case study, we suggest that the presented framework has the potential for paving the way to more integrated studies of whole organisms leading to a systemic analysis of cells on a broader scale. The modeling of other organisms using this framework could provide useful large-scale models for different fields of research such as bioengineering, network biology, and synthetic biology, and also provide novel tools for medical and industrial applications.

## Introduction

Cells are mainly composed of water, proteins, nucleic acids, metabolites, and an enveloping lipid membrane. However, what makes a cell alive are the interactions between these components. Chemical reactions interconnect molecules into intricate biochemical networks in order to perform particular tasks. The whole set of possible chemical interactions is meticulously regulated in order to maintain cellular function, growth, and replication^1^.

Biochemical interactions with related functions have been traditionally grouped into cellular processes^2,3^. Among these processes, metabolism^4,5^, signaling^6–8^, and transcription regulation are those most frequently described and modeled as networks^9–12^. Though cellular processes are often described in terms of separate networks^13,14^, they are neither physically nor functionally independent^15^. The simple fact that molecular species are shared between them makes their dynamics dependent on each other. For instance, the intracellular concentration of the energetic molecule ATP affects and is affected by, several processes simultaneously.

The ever-growing data availability regarding cellular biology has implied in ever more comprehensive computational models of cells^16,17^. Having pursued metabolic, signaling, and gene regulation networks toward the limit, we are now interconnecting these data^18–21^. Bolder approaches are aiming at complete representations of unicellular organisms^22–24^, taking into account all known cellular processes, whether it is network-oriented or not. For example, Karr *et al.*^23^ performed whole-cell simulations of the organism *Mycoplasma genitalium* using a hybrid simulation approach. Briefly, cellular processes are simulated independently using diverse simulation approaches, and information is interchanged only from time to time. Such whole-cell approaches are paving the way to enhancing the pace of advances toward the understanding of cellular behavior, favored by their capacity to predict cell’s phenotype from genotype with good accuracy^25^.

Despite the promising achievements of the above mentioned whole-cell approaches, much remains to be done towards achieving reference modeling frameworks^26^. Current models are tailored for respective organisms, leaving no straightforward means to adapt them for other species. The hybrid approach to modeling and simulation also has its limitations. Additionally to integration issues in simulations, which are out of scope for this work, it is difficult to have a broader view of the biological system of interest once relationships between molecules are encapsulated within several distinct mathematical and computational modules. At the same time, it is not an easy task to describe such modules using community standards such as SBML^27^.

Assuming that all cellular processes can be approached by their underlying biochemical interactions, we can ultimately integrate them by using reactions that share the same cellular substrate, such as given proteins, DNA, and metabolites. Thus, there should be an approach allowing all cellular components and known interactions to be accommodated into a single biochemical network. Such a framework could then provide a whole picture of the possible molecular interactions inside a cell at any time of a cellular cycle. Moreover, the role of given molecules could then be addressed in a systemic context, revealing interfaces between cellular processes.

In this work, we aim at modeling and integrating several cellular processes into a single biochemical network, hence providing a more homogeneous framework to model whole organisms. We propose a rule-based modeling approach to represent a diverse variety of interactions between molecular species that can be found within a cell. The set of molecules and reactions composes a biochemical network where molecules are linked to reactions according to their role as reactants or products. In addition, we explicitly link to reactions the molecules that act as their catalysts or regulators. For the sake of generalization, we hence call this kind of relationship as *modifiers*, following the nomenclature already implemented in SBML^28^. The stoichiometry of the interactions is described as weights associated with the links. To model cellular processes that are not usually represented as networks, we built sets of reactions (templates) that can be replicated for different substrates such as the transcription of several genes, or the translation of several mRNAs.

As a study case, we modeled biochemical and genomic data about all the known processes of the bacterium *Mycoplasma genitalium* resulting in what we call a “whole-cell biochemical network” of the organism. We choose this organism because of its relative simplicity, corresponding to the smallest known genome, and for having respective integrated data at the whole-cell level deposited in the WholeCellKB^16^. The network contains information about 15 cellular processes, naturally integrated into a single component. To validate the model, we perform cascading failure analysis to predict essential genes of *M. genitalium* and compare the obtained results with experimental data from global transposon mutagenesis gene disruption^29^.

We believe that the presented approach can pave the way to more scalable and adaptable whole-cell models while providing a more homogeneous basis for whole-cell analysis and simulations. Other interesting prospects could be explored in the context of constraint-based methods^30,31^, synthetic biology^32,33^, and network science^34–36^.

## Modeling cellular processes through reactions

All cellular processes can be understood by means of their underlying biochemical reactions. When trying to do so, one can face two related problems: the lack of sufficiently detailed information and the highly combinatorial nature of biomolecular interactions. By far, the most detailed cellular process in terms of specific biochemical reactions is the metabolism^37^. For many organisms, we already have very detailed and maybe complete metabolic maps^38^. Even though, metabolism is just a small set of reactions among many others that composes other cellular processes. One step further from metabolic interactions we can find their proteic catalysts, known as enzymes. Proteins such as enzymes can often be modified in specific sites, therefore modulating their activity as catalysts and also their interaction with other proteins. The number of different states for each protein in the organism and the complexes they can form grows very fast thus bringing us to the combinatorial problem.

One approach to model the high number of states and interactions of proteins is the rule-based modeling^39^. In brief, this method makes use of generic rules representing molecular interactions that can be applied to many substrates. By doing so, there is no need for the modeler to manually write all the possible interactions among substrates, which can be now automatically generated by the so specified rules. Rule-based modeling is mainly applied to study the signaling process in cells^40,41^. The high number of protein modifications and complex formations lead us to the first-mentioned problem: the lack of detailed information regarding which of these interactions really occurs.

While the interaction of metabolism and protein-protein interactions have already been tackled in the literature^42^, other cellular processes such as protein and RNA synthesis, DNA replication, protein and RNA degradation, and cell cycle, are rarely integrated to these models at the biochemical level. Thus, in the next sections, we will make use of the rule-based modeling principles allied to whole-cell scale databases to propose representations of the most diverse type of molecules and interactions possible of given organisms. Our approach aims at expanding the rule-based modeling to encompass all known cellular processes integrating them into a single reaction network.

### Reaction modeling framework

**Figure 1:**
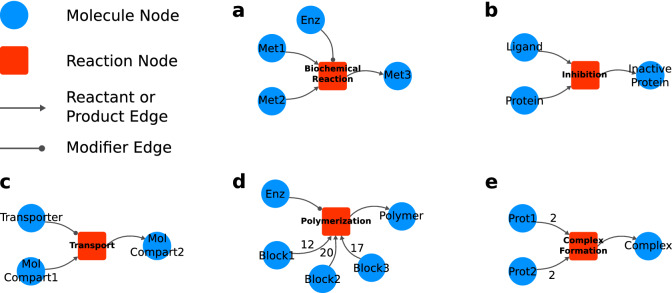
Graphical representation of the reaction modeling framework. Molecule nodes are represented by blue circles. Red squares represent reaction nodes. Arrow-ended edges indicate reactant or product relationships depending on direction. Circle-ended edges indicate modifiers to the reaction. Some modeling examples of distinct kinds of biochemical interactions are depicted as follows. (**a**) A biochemical reaction ${\hbox {Met1} + \hbox {Met2} \xrightarrow {\mathrm{Enz}} \hbox {Met3}}$; (**b**) inhibition of a protein by a ligand; (**c**) molecular transport from compartment 1 to compartment 2 by a transporter; (**d**) a polymerization reaction; (**e**) a tetrameric protein complex formation. All detailed network visualizations in this and further Figures were generated using Cytoscape^43^.

To better explain our modeling approach, a simple graphical notation will be used to depict molecules and interactions in the form of a network. Thus, using network science nomenclatures, representations of molecules and reactions will be called as *nodes*. Relationships between molecules and reactions will be called as *edges*. Figure 1 shows the graphical notation adopted in this work.

In our framework, molecule nodes can represent any physical entity within a cell such as metabolites, DNA regions, proteins, and RNAs. Each different state of a molecule (e.g., the active and inactive form of a protein) will be represented by different molecule nodes. Similarly, molecules in different cellular compartment locations (e.g., intra and extracellular glucose) are also represented by distinct nodes.

We can represent any interaction between molecules by a reaction node. In addition to biochemical reactions, such as in metabolism, more complex interactions such as gene transcription, protein synthesis, transport, protein complex formation, chromosome replication, and cell division can be incorporated in the model.

Reactions can be regulated and we can explicitly link the “modifiers” to their respective reaction nodes. To do so, we use a distinct type of edge, called *modifier edge*. In Fig. 1a, the modifier edge is drawn as a circle-ended line connecting molecule “Enz” to the reaction. This connection means that the Enzyme is needed so that the reaction can occur, but it is neither consumed nor produced in the reaction. For example, other molecules such as transcription factors, genes, and mRNAs can act as modifiers, since their concentration does not change in some reactions.

To illustrate the modeling of some molecular interactions, Fig. 1 shows some use cases derived from the generalizations we adopted. Example (a) depicts a biochemical reaction where an enzyme combines Met1 and Met2 into Met3. In (b), a given protein interacts with a ligand (e.g., in an allosteric site) producing the protein’s inhibited form. Transport through a cellular membrane can be approached as in example (c), where a given molecule is carried from extra to the intracellular environment by a transporter protein. Example (d) illustrates a polymerization process catalyzed by an enzyme where different stoichiometries of three basic building blocks are combined into a single polymer, such as in protein synthesis reaction catalyzed by a ribosome. Protein complexation of four subunits (e.g., $\alpha$ and $\beta$ hemoglobin subunits) is represented in example (e).

### Process templates

Certain cellular processes make use of the same molecular machinery to produce different outputs given different inputs. It is the case of the synthesis of macromolecules such as proteins, RNAs, and DNAs. For instance, the synthesis of proteins involves a set of reactions that repeats for each mRNA given as an input. Most of the molecules involved in the process are the same for different mRNAs, sometimes only changing the stoichiometry of amino acids. For such processes, we created templates which are sets of reactions that can be replicated and adapted for different substrates in the same sense of rule-based modeling. Particularities of each reaction can be incorporated if data is available. It is important to notice that the templates generated in this work are made specifically to the organism used as an example and the modeling assumptions can vary from modeler to modeler.

## *Mycoplasma genitalium* case study

Recently, a public database called WholeCellKB was implemented aiming at gathering complete biological information about specific organisms^16^. The first organism deposited in this database was the pathogenic bacterium *Mycoplasma genitalium*. This organism yields the smallest genome known, with 580 kb and 525 genes. Because of its relative simplicity, *M. genitalium* has served as the model organism for breakthrough advances in synthetic biology^29^ and whole-cell simulations^23^. The simplicity of this organism, allied to the structured data provided by the database, makes *M. genitalium* a particularly suitable model for the comprehensive integration of cell-scale biochemical interaction into a whole-cell biochemical network.

Using the proposed framework, we modeled almost all the biochemical interaction information contained in WholeCellKB about *M. genitalium*. The database accounted for interactions for the following processes: DNA Replication Initiation and Elongation, DNA Damage, DNA Repair, Cellular Division, Transcription, RNA Processing, tRNA Aminoacylation, Translation, Protein Modification and Complexation, Metabolism, Transmembrane Transport, Host Interaction, and RNA and Protein Decay. The only process not taken into account was the DNA Damage, which we could not find an appropriate means to represent in our model because of its high combinatorial nature. Also, RNA modification reactions were not incorporated in the model due to systematic inconsistencies found in the WholeCellKB regarding these reactions. Some processes described in Karr’s work^23^ were encompassed by major processes in our model. For example, processes like “Protein Folding” and “Protein Processing” were incorporated by “Translation”. The “Ribosome assembly”, and “FtsZ ring polymerization” were incorporated by ‘Macromolecular Complexation”. The “Chromosome condensation” was incorporated by “Protein-DNA Interaction”. The “Chromosome segregation”, and “Cytokinesis” were incorporated by “Cellular Division”.

**Figure 2:**
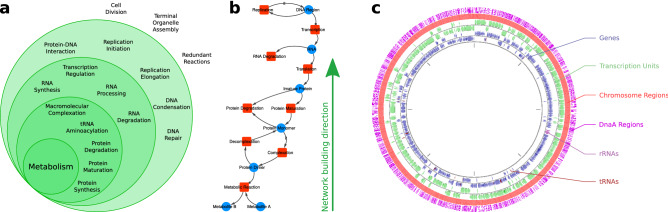
The hierarchical construction of the *Mycoplasma genitalium*’s whole-cell biochemical network. Starting from the metabolism several cycles of introducing necessary molecules to the reaction and their synthesis/degradation reactions. A simplified version of a full cycle of network building starting from a single metabolic reaction is depicted in **b**. When the network construction reach the DNA level, the chromosome is divided in regions in order to better represent locus-specific protein interactions. Figure **c** was partially generated using CGView^44^.

### Network building process

To start building the biochemical network of the *M. genitalium* organism, we first queried all metabolic reactions from WholeCellKB and included them as reaction nodes in the model. Then, all metabolites which act as reactant or product, as well as enzymes, were incorporated in the network as molecule nodes and properly linked to their respective reactions. From this point, a recursive process starts by adding biosynthesis and degradation reactions for each molecule already in the network. For example, protein complexes are biosynthesized by macromolecular complexation reactions. Protein monomers are produced by translation and protein modification reactions. They also are degraded by proteolysis. For these newly incorporated reactions, the necessary molecules are added as molecule nodes and linked to them. This process repeats itself until all molecules have their respective biosynthesis and degradation reactions. At the end of this recursive process, it is expected the network to have all reactions from metabolism to DNA Replication, passing through all the central dogma of biology (Fig. 2a). A simplified example of a full pathway from DNA to metabolite is depicted in Fig. 2b a homodimeric enzyme that catalyzes a given metabolic reaction has its biosynthesis pathway built upwards to the DNA level passing through protein complexation, protein maturation, protein synthesis, RNA synthesis, and DNA duplication. Degradation reactions for proteins and RNA are also shown.

On top of the so obtained network, we queried the WholeCellKB for reactions that are still not included in the model, such as redundant reactions, and added them. To finalize the network, we manually included the “Cell Division Reaction” to which all necessary proteic complexes, such as FtsZ Ring, Chromosome Segregation proteins, and the duplicated Chromosome, are linked as modifiers. A detailed description can be found in the Supplementary Information.

In order to guarantee the coherence of the reactions in the network, we calculate the mass-balance for all reactions. Following the principle of mass conservation, the difference between the mass of reactants and products, weighted by their respective stoichiometry, should be zero. Although this approach can assure the mass-balance, literature evidence is still needed to ensure their correctness.

### Chromosome representation

Despite being the smallest known chromosome, *M. genitalium*’s still a large and lengthy circular molecule having 580kb. In order to better represent locus-specific protein interactions, we divided the chromosome into regions, each one being represented by a molecule node.

We used as reference the *M. genitalium* G37 genome^45^, available at the NCBI database (NC_000908.2). In Fig. 2c we can observe the circular chromosome representation as well as the genes distributed along with it. The transcription units (TUs) are the regions that are transcribed in RNA. One TU can encompass one or more genes, the last case also being called “Polycistronic RNAs”. The RNAs from TUs with more than one gene can be further cleaved into separated RNA molecules, which is the case of tRNAs, or left as one molecule. In any case, each RNA molecule, polycistronic or not, is represented by a single molecule node.

Although intuitive approaches would be to divide the chromosome regions according to the TUs or even to split into regions of the same length, the more granular the division, more details can be incorporated in the model. The DnaA protein interacts with small  8 nucleotides length sequences repeated all over the chromosome. Figure 2c depicts the distribution of DnaA binding sites annotated in the WholeCellKB. The binding and polymerization of this protein at specific nucleotide sequences in the chromosome are the main mechanisms to control cellular replication and the binding sites present a more granular division of the chromosome. Thus, we adopted the DnaA binding sites as division points to define the chromosome regions, with the addition of the replication origin and terminus sites. More specifically, each DnaA biding site is defined as a chromosome region, and each region between DnaA binding sites is also defined as a chromosome region.

### Modeling canonical processes

Although some processes, such as metabolism, have a straightforward modeling transition from WholeCellKB to the proposed framework, other processes require more attention. For instance, it is not usual to describe chromosome replication, gene transcription, protein synthesis, and some other processes as networks. Thus, we manually created templates based on literature for these processes. Particularities in the synthesis process of individual proteins, RNAs, and DNA are incorporated accordingly to data availability in WholeCellKB.

#### Chromosome replication

The chromosome’s replication starts when the DnaA protein polymerizes in five specific DnaA binding regions near the replication origin and recruits all necessary molecular machinery to replicate the DNA. It is the formation of the two Replisomes at the origin of replication in the chromosome, which we call the Replication Complexes.

**Figure 3:**
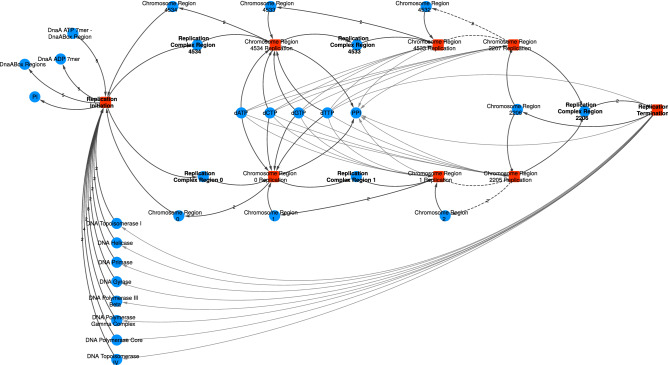
The template for Replication Initiation and Replication Elongation processes. The network depicted shows three steps of Replication Elongation going both chromosome directions. The dashed lines mask the rest of the process and go straight to the final reaction, where the terminus region (2206) is replicated and the Replisomes released. The actual molecules’ IDs in the network are listed in the Supplementary Information. Although some stoichiometries are shown, most of them are hidden for better visualization.

Given that the chromosome is divided into regions, the formation of the Replication Complex also takes as a reactant a chromosome region. The two Replisomes, bound to the Chromosome Regions 0 and 4534, undergo their respective replication reactions where the free deoxy-nucleotides are consumed according to the regions’ sequence. Each replication reaction produces two copies of the current Chromosome Region and consumes the next region, making the Replisome move through the DNA molecule (Fig. 3). The two Replication Complexes move in opposite directions until they reach the replication terminus region, where the replication completes and the Replisomes’ subunits are released. The collision of the Replisome with other Protein-DNA complexes, such as DnaA and RNA Polymerases, are handled separately.

#### RNA synthesis

**Figure 4:**
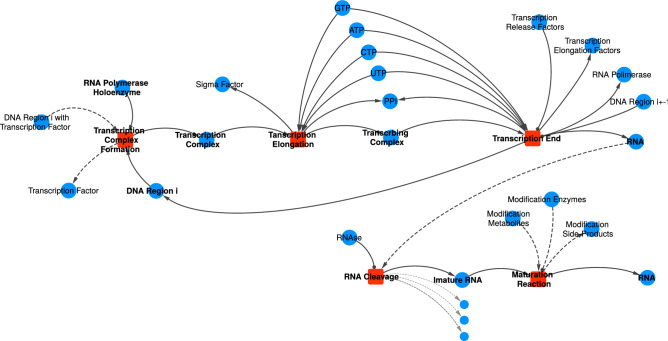
The template for the Translation process. Dashed lines indicate relationships that are not always required. For example, not all RNAs goes through RNA Cleavage and Maturation reactions, such as tRNAs. Most of the RNA modifications available in WholeCellKB were not included in the model due to systemic inconsistencies found in the database regarding the positions of the modifications.

The RNA Synthesis is the process in which an RNA Polymerase makes an RNA molecule based on a Transcription Unit (TU), a region of the chromosome that may contain one or more genes. Given that a TU can be fragmented in several Chromosome Regions, as observed in Fig. 2c, the transcription process follows a similar approach to the Chromosome Replication, once the Transcription Complex moves through the DNA and its template is shown in Fig. 4.

The binding of the RNA Polymerase Holoenzyme to the beginning of a TU in the chromosome might require a Transcription Factor already bound to the respective Chromosome Region, as indicated with dashed links to the “Transcription Complex Formation” reaction in Fig. 4. Then, given that the TU begins at the Chromosome Region *i*, the Transcription Complex, the example in Fig. 4 depicts a TU divided into only two Chromosome Regions, being the second one at the position $i+1$ or $i-1$ depending on which strand of the DNA the TU is found. TUs that are polycistronic and need to be cleaved to produce the individual functional molecules, the so transcribed RNA goes through further cleavage and maturation reactions.

#### Protein synthesis

**Figure 5:**
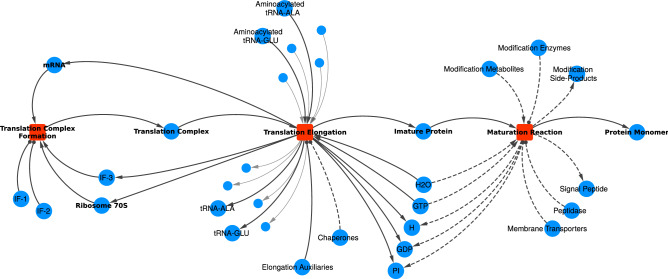
The template for Translation and Protein Maturation processes. Dashed lines indicate relationships that are not always required. For example, not all proteins require Chaperones. Membrane Transporters, Peptidase, and the production of the Signal Peptide are only required for secreted proteins. Only two of the twelve amino acids are shown to better visualization. The small blue circles illustrate the other amino acids and associated tRNAs.

The template for the translation process is shown in Fig. 5, including the translation complex formation, translation elongation, and protein maturation.

The translation process starts with the complexation of the Ribosome 70S, Initiation Factor (IF) 3, and the mRNA with IF-1 and IF-2 as auxiliary molecules. Then, the translation complex proceeds to the elongation stage where aminoacylated tRNAs and energy molecules (GTPs) are consumed according to the mRNA sequence while the respective tRNAs without amino acids and the IF-3 are released. Elongation auxiliary proteins act as modifiers and chaperones might also be linked depending on the protein’s annotations in WholeCellKB. Similarly, post-translational modifications can be incorporated in the Maturation Reaction, transforming an immature form of the protein into the functional one. Proteins that are secreted to the external environment have also linked to the Maturation Reaction the necessary membrane transporters, the peptidase to cleave the Signal Peptide, and the Signal Peptide itself.

A more detailed description of the translation’s modeling, including exact labels and nomenclature adopted for nodes, as well as the modeling templates for other processes, namely Transcription Stall, Translation Stall, Protein and RNA Decay, and Cellular Division, can be found in the Supplementary Information. Also, one should notice that all the following analysis refer to the network built using our modeling assumptions. Different modeling assumptions might lead to different structures, and therefore, to different conclusions.

## Cascading failure analysis

Some specific gene deletions can trigger a deadly cascade of failures in cells. Other deletions might not cause such an impact, characterizing cellular robustness^46^. Cascading failure analysis has been used to evaluate robustness on several network-based systems^47–50^. Analogously, it has been successfully applied for the estimation of essential genes in metabolic networks. They removed reactions regulated or catalyzed by a given gene product, and the impact of cascade failure propagation on the network structure revealed a correlation with gene knockout lethality^51,52^.

The proposed framework and metabolic networks share many structural characteristics. Because of their similarity, the same cascading failure approach can be used to estimate the impact of gene deletion on a whole-cell biochemical network.

**Figure 6:**
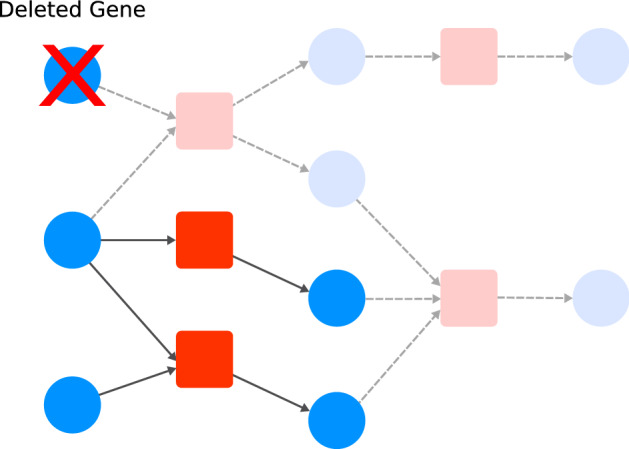
Example of cascading failure. The nodes with shaded color and dashed edges were removed after cascading failure by removing the initial gene molecule node.

We adopted the same algorithm described by Mombach *et al.*^51^ to perform the cascading failure analysis. As demonstrated in their work, cascade failures were started by removing reactions regulated by a given enzyme in order to quantify its essentiality. However, enzymes and other regulators are explicitly connected to their respective reactions in our framework. Thus, we use molecule nodes which represent the functional molecule of each gene as starting points of the cascade failure dynamics, instead of reaction nodes, as shown in Fig. 6. For genes that are further translated into proteins, we selected the molecule nodes, which represent its protein monomers. For tRNAs and ribosomal RNAs, the selected nodes were the ones representing the RNA molecules themselves.

In order to observe the effect of gene deletion in the network, we checked if a specific critical reaction, the cellular division, is still present in the network after the gene deletion. In other words, we remove the node representing the respective gene and perform the cascading failure analysis. Then, the gene is classified as essential if the critical reaction is absent in the network. Otherwise, the gene is classified as non-essential. Additionally to single gene deletions, we performed double gene deletions to analyze relationships between pairs of genes.

### Network rewiring

Randomization of network topologies is one of the most commonly applied methods to investigate how much information is encoded within a model^53–55^. Here we employ a randomization process that changes the source and target of each edge with a probability *p*. For each edge, a random number between 0 and 1 is generated from a uniform distribution. If this number is smaller or equal than the probability *p*, the source and target of this edge are assigned to new nodes randomly chosen. The new nodes are chosen among ones with the same type to keep the bipartite characteristic of the network.

## Results

**Figure 7:**
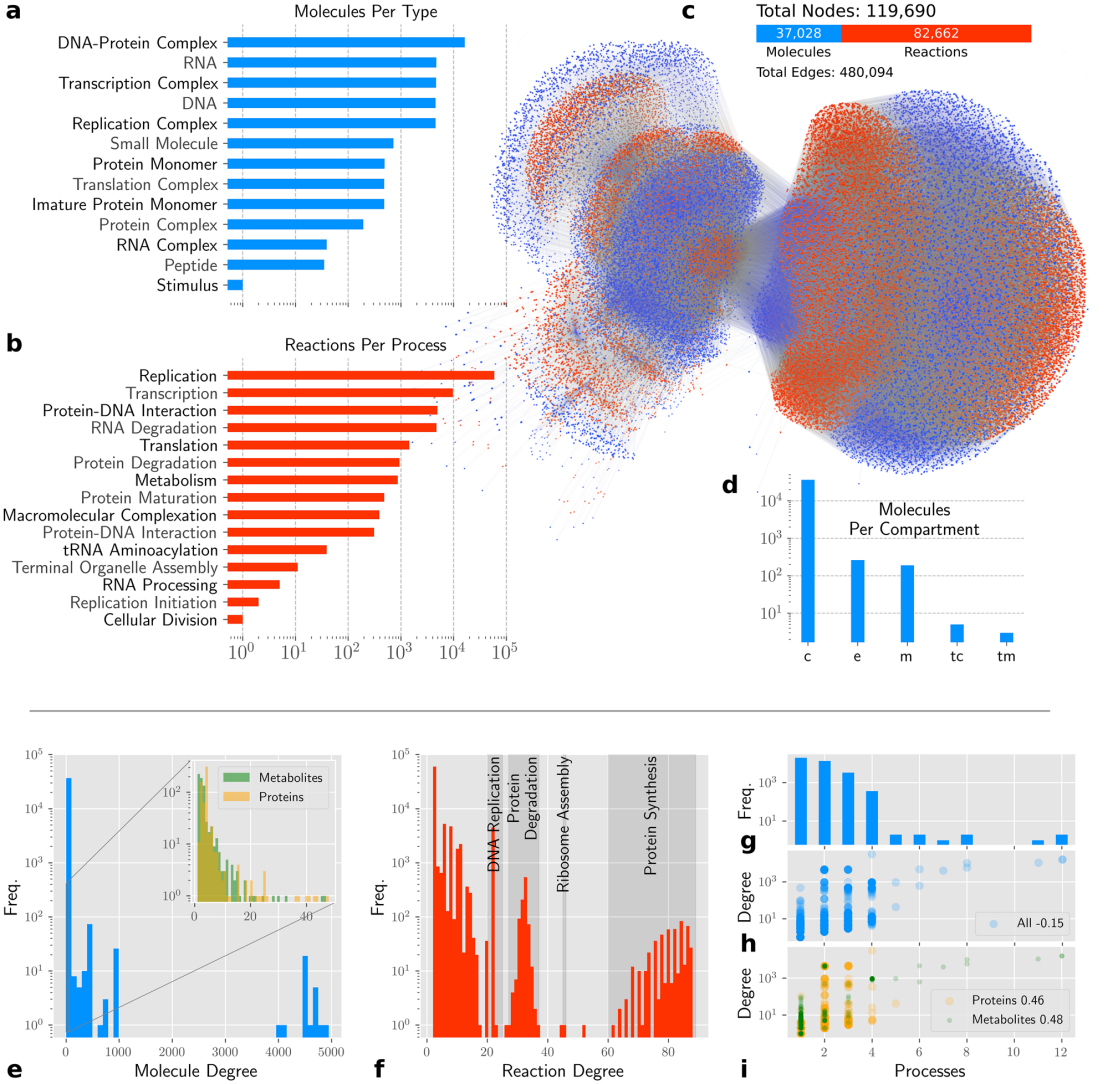
The *M. genitalium* whole-cell biochemical network an topological analysis. **a** number of molecules per functional group in logarithmic scale. **b** Number of reactions per cellular process in logarithmic scale. **c** Graphical visualization of the *M. genitalium* whole-cell network. Edges with high associated stoichiometry (over 100) are hidden for better visualization. The big circular group on the right is mainly composed by DNA-Protein complexes and their respective formation reactions. **d** Number of molecules per compartment in logarithmic scale. **e** Degree distribution of molecule nodes. The subgraph in the figure is the same degree distribution but showing only the protein and metabolite subgroups of nodes. **f** Degree distribution for reaction nodes where de degree is the number of connections solely. **g** Distribution of number of processes a given molecule node participates. **h** Spearman correlation between node degree and number of processes for all molecule nodes. **i** Spearman correlation between node degree and number of processes for only proteins and metabolites.

Based on WholeCellKB’s^16^ and genomic information, we built the whole-cell biochemical network of *M. genitalium*, comprising the molecular types and cellular processes indicated in Fig. 7a,b.

### Topological analysis

The generated model is a bipartite, weighted, and directed network containing a single connected component with 119,690 nodes and 480,094 links (Fig. 7c). The nodes comprise 37,028 molecules and 82,662 reactions (Fig. 7c). The network is available in GML and SBML formats in a Github repository indicated in section “Availability of Data and Materials”. We annotated molecule nodes with their functional group. Figure 7a shows its distribution among the nodes. We also annotated reactions according to the cellular process to which they belong. As shown in Fig. 7b, almost all known cellular processes can be found among the reaction nodes.

Figure 7d depicts the distribution of nodes between three considered locations: cytosol (c), cellular membrane (m), Terminal Organelle Cytosol (tc), Terminal Organelle Membrane (tm), and extracellular environment (e). The 249 molecules located in the extracellular environment account for nutrients, side-products expelled from metabolism as well as secreted proteins.

Considering the *degree* of a node as the number of connections it owns, we analyzed the degree distributions for molecule and reaction nodes. For both cases, multi-modal distributions were obtained. It is known that metabolic and protein-protein interaction networks often have power-law-like degree distributions^2,4,56,57^. Thus, we analyzed the distribution of proteins and metabolites separately (Fig. 7e). We found that their distributions corroborate literature, but this assumption is not true for the whole system. The degree distribution for reactions showed different well-separated distributions that were found to be related to different processes (Fig. 7f). For instance, Protein Degradation reactions were found grouped in a Gaussian-like region. Other processes have reactions with a signature degree, such as DNA Replication and Ribosome Assembly, the former being accounted into the Macromolecular Complexation process.

Regarding the network completeness, 20% of the proteins monomers and protein complexes (a total of 137 molecules) have no interactions described in the WholeCellKB. These molecules are connected only to their biosynthesis and degradation reactions, participating in no other interactions. Many of these molecules with unknown function are putative membrane proteins. Because there is still no signaling pathway reported in *M. genitalium*, these putative membrane proteins can be a starting point for the elucidation of signaling processes in this organism.

### Interface between processes

Molecules shared by reactions from different processes are interfaces between them. It means that the concentration of such molecules can affect, and be affected by, the dynamics of more than one cellular process. We found that 46.8% of the molecule nodes participate in at least two cellular processes, making the bridges between them. Parting from the hypothesis that the more connected a molecule is in the network, the higher is the chance that it connects cellular processes, a positive correlation is expected between the node’s degree and the number of processes that it participates. Figure 7h shows that it is not the case, where we obtained a low negative correlation between those measures. Nevertheless, we found a significant positive correlation when analyzing only proteins and metabolites (Fig. 7i).

### Gene essentiality prediction

In order to validate the *M. genitalium* whole-cell biochemical network generated in this work, we simulated gene deletions by performing the removal of nodes that represent each gene product, followed by cascade failure, and then analyzed the damage caused on the network. The simulation results regarding the 525 genes of *M. genitalium* were compared to experimental gene essentiality classification by global transposon mutagenesis gene disruption available in the literature^29^. The experimental data classified 382 genes out of 525 as being essential to the organism so it can replicate itself.

We performed single and double gene deletion experiments. For single-gene deletions, each gene was removed following by its cascading failure. Table 1 shows the comparison with experimental data achieving 54% of exact matches with 1.7% of false positives.

**Table 1:** Validation of gene essentiality predictions against experimental data.

	Match	False positive	False negative
Single	0.5409	0.0171	0.442
Double	0.56	0.0190	0.421
Only essential	0.9524	0.0476	0.0

**Figure 8:**
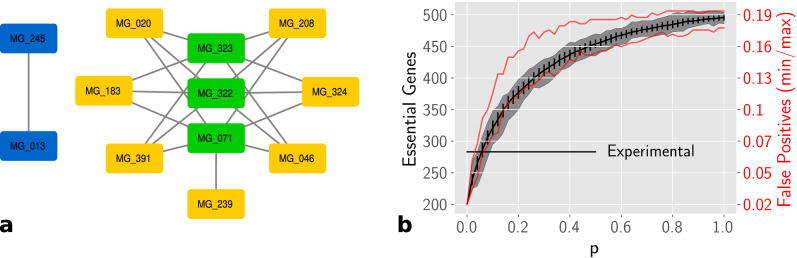
**a** Genes classified as essential in double deletions. The connected pairs means that their conjunct deletion resulted in the deletion of the cell division reaction. They are grouped by colors respective to their functional groups: cross-membrane ion transporters (green), protein degradation (yellow), and folate metabolism (blue). **b** Gene essentiality prediction of randomly rewired networks with probability *p* from the original *M. genitalium* whole-cell biochemical network. Each rewiring rate accounts for 50 replicates.

For double gene deletion, we simultaneously removed each pair of genes and performed the cascading failure. The double gene deletion slightly enhanced the classification, which resulted in 56% of correct matches. Among the genes classified as essential only in the double deletions, three distinct groups could be outlined as depicted in Fig. 8a. The first is composed of three genes (MG071, MG322, and MG323) responsible for ion transport across the membrane. The second group is composed of seven genes (MG020, MG046, MG183, MG208, MG239, MG324, and MG391) involved in the protein degradation process. The deletion of almost any combination of genes from these two groups, except for MG239 that is only essential with MG071, indicates a non-viability of the cell. The third group is composed of two genes (MG013 and MG245) that are involved in the folate metabolism and are only classified as essential if both are simultaneously removed.

It is important to observe that genes with unknown functions, which compose 22.24% of all genes, were included in this analysis. These genes might have a considerable impact on the matches rate, once all were classified as non-essential. Nonetheless, considering only genes classified as essential by the combined approach, the correct matches increase to 95%, indicating the high accuracy of the model.

In order to check the statistical relevance of the *M. genitalium* whole-cell biochemical network, we generated randomly rewired networks based on the original one by reconnecting each edge, preserving directionality, with a probability *p*. A total of 50 replicates were generated for each probability with *p* ranging from 0 to 1 with a 0.02 step. In Fig. 8b we can observe that the higher the network randomization level, the higher is the number of false positives in the essentiality classification.

## Discussion

### *Mycoplasma genitalium* whole-cell biochemical network

We choose the pathogenic bacterium *M. genitalium* organism as a case study to illustrate the capabilities of the proposed framework because of its simplicity and data availability in the WholeCellKB database. Despite its simplicity, the so obtained network comprises thousands of biochemical interactions from which several cellular processes emerge.

This approach provides a means such as that all cellular processes are explicitly described as a unified network of biochemical interactions. To support this affirmation, we mention the fact that the whole-cell biochemical network of *M. genitalium* has only one component, which means that all biochemical interactions are in some level interconnected. Moreover, 46.8% of the molecules participate in reactions respective to more than one process, acting as the intrinsic interfaces between them. Despite the analyses performed in this work, we believe that more information about how processes are integrated could be extracted from this model.

Indications about the reliability of the obtained reactions can be derived from the WholeCellKB, where each reaction is assigned to its respective literature evidence. The reactions in the network that were not explicitly described in the database, such as replication, transcription, and translation reactions, were derived from basic biological knowledge about these processes. Some of these reactions also incorporated particular molecules such as transcriptional factors and chaperones, which therefore have their literature evidence indicated in the database as well.

### Network topology

Network models of natural systems usually share some topological characteristics. Considering the *degree* of a node as the number of connections it owns, the degree distribution through the nodes of a network is one of these shared features^58^. Somehow, it is believed that the processes that create these natural systems, particularly the biological systems, resulted in patterns of interaction between its elements which follows a power-law distribution^2,4,56,57^. In other words, these systems tend to have several elements with few interactions and some few elements with a high number of interactions. This feature is one of the current explanations for the robustness of living systems^46,59,60^. For example, if we consider a random failure among its elements, we will find that it is more likely the failure to affect elements with few connections, which tend to have more secondary than central roles in the system.

We found evidence that the degree distribution in our network resembles a power-law but only for selected molecule groups. It is the case of proteins and metabolites. The degree distribution when considering all the nodes is not trivial. Whilst having found such power-law-like structures inside the network, they represent a small fraction of all molecular entities represented in the model. The assumption of a cell’s robustness explained by network topologies seems more limited in this perspective, even though the so obtained distributions rely on our modeling assumptions. Nonetheless, the properties of the power-law-like subsets could propagate to the rest of the network by cascading failures.

### Essential genes

As a validation of the so obtained model, we simulated gene deletions by removing nodes that represent gene products, followed by their respective cascading failure, to analyze the impact of their removal on the network. High impact removals were associated with gene essentiality and compared to experimental data. Considering the static nature of the network, and the fact that 22% of the genes have unknown function, a reasonable result was obtained, achieving 56% of correctness in gene essentiality prediction. The high rate of false negatives can be a consequence of several reasons. Firstly, not all genes have a known function. Secondly, even if the deletion of a gene does not cause significant damage to the network structure, the cell may become nonviable for dynamical reasons, such as flux bottlenecks in metabolic pathways that cannot be captured in our analysis. It is supported by the fact that almost no genes directly related to metabolism were classified as essential. Also, a given deleted gene classified as non-essential could perhaps provide the cell with the ability to grow in specific environmental conditions considered in the experimental analysis.

An interesting observation is that no significant improvement, approximately 2%, was obtained from single to double gene deletions, suggesting little redundancy in the genes with at least one known function. Therefore, we may outline the following hypothesis while considering that *M. genitalium* has a restricted genome size: (1) the organism achieves robustness through other means; (2) there are genes which have currently unknown function which could provide such redundancy; or (3) genes with at least one known function might have more roles in the cell.

Despite little improvement in the prediction, double deletions provided useful insights into *M. genitalium*’s biology. For instance, the fact that the single deletion of trans-membrane ion transporters does not have an impact in the cell division reaction, suggests that the cell has other means to obtain those ions. This other mean could be inferred by the double deletion of transporters together with genes related to protein degradation. In other words, the degradation of proteins inside the cell can provide recycled ions to the cell’s cytoplasm.

Although we could not classify all of the 382 genes experimentally identified as essential, 95% of the genes we did classify as essential were correct. We can then assume that we cannot rely on the correctness of our approach if a gene is classified as non-essential, however, if a gene deletion imposes significant damage to the network, it has 95% chance of being essential to the organism indeed. Furthermore, our approach provided a computationally inexpensive solution to identify primordial genes to the cell’s reproduction, being able to be computed within a few minutes on a regular computer.

Additionally, rewiring simulations showed that little changes in the network’s topology increase the number of genes classified as essential but also the rate of false positives, suggesting that the results of gene essentiality prediction are strongly related to the network structure.

### Related modeling methodologies

Network-based models have been extensively used to represent cellular processes. Besides to metabolic and signaling networks, which was already tackled in this work, we can give as examples: (a) genetic regulation network, representing positive and negative relationships between gene expressions^61,62^; and (b) protein-protein interaction (PPI) networks, indicating physical interactions between proteins^63–65^. Some of these networks, namely metabolic and signaling networks, are already representing their processes based on their underlying biochemical reactions. On the other hand, genetic regulation and PPI networks represent processes that are composed sometimes by several intermediate biochemical reactions, therefore providing a high-level representation.

If we consider a set of networks representing processes of the same organism by using the network models above mentioned, there is currently no means for a straightforward integration between them once they have very different modeling assumptions. Studies addressing the integration of cellular processes often maintain the respective underlying networks in separated layers^19,20,66,67^.

No quantitative comparison can be established between the so constructed *M. genitalium* network and any other network model for two reasons: (a) there is no other network model for this organism; (b) no modeling approach have integrated cellular processes that could be topologically addressed as a single network. Nevertheless, the closest approach available in the literature is the one provided by *Reactome.org*^17^. They aim at representing several different cellular processes through biochemical reactions. However, biochemical pathways are organized in a nested form, thus not providing a single integrated network. Also, processive processes, such as transcription and translation, are not represented. Even so, *Reactome.org* represents an accessible, curated, and annotated biochemical content which can be further transcribed, with no much effort, into single whole-cell networks using our approach.

Regarding the network’s construction process, our approach could further benefit from related methodologies for metabolic networks. For instance, orthology-based network reconstructions^68,69^ could be directly applied using the *M. genitalium* whole-cell biochemical network and other networks of model organisms that can emerge. The development of databases containing whole-cell biochemical networks would be increasingly useful for such reconstructions as new models are provided, also benefiting the study of non-model organisms.

## Conclusions

In this work, a framework was presented for integrating cellular processes at a whole-cell scale, using rule-based modeling in a broader context. Besides the incorporation of network-modeled processes, such as metabolism, we were also capable to model processes that are not usually represented as networks, such as replication, transcription, and translation. We applied the framework directives to model whole-cell scale information about the *Mycoplasma genitalium* organism stored in specialized databases aiming to probe the capabilities of the framework. The obtained whole-cell biochemical network accounted for a great variety of molecules and cellular structures, as well as the interactions between them covering almost all processes known in the organism.

Many are the applications of whole-cell biochemical networks. In bioengineering, for example, it could provide more extensive biochemical interaction maps of given organisms, serving as a tool to better manipulate them. Also, the overlapping between whole-cell biochemical networks of interacting organisms could provide insights on their relationship at the molecular level. Whole-cell biochemical networks can also pave the way to more comprehensive complex networks-based investigations, where we could study whole organisms through the topology of their biochemical interactions in a broader sense.

Whole-cell biochemical networks could also provide plenty of information for constraint-based approaches such as Flux Balance Analysis (FBA). If not considering modifier edges, the whole-cell metabolic network has the same structure of metabolic networks, therefore being able to be represented by a stoichiometric matrix. Regulation relationships indicated by modifier edges can be incorporated in FBA as additional constraints to their respective flux’s boundaries. Networks generated with the proposed framework can also serve as the underlying model to deriving dynamic simulations using approaches such as the reaction rate equations and the stochastic simulation algorithms.

Despite the promising applications of the framework, the current scripts are limited to read data only from the WholeCellKB. Though impressive amounts of biological data continue to be generated, they tend to flow into relatively specific analyses and databases. A next step would be to develop software capable of searching these databases, integrate information from different sources, and therefore provide a more comprehensive and automated approach to whole-cell modeling. Some initiatives are already heading in the direction of aggregating available data^70^, making it usable for simulation purposes, and performing community-based development and validation of whole-cell models^71^. In this sense, our framework could pave the way for community-based modeling, where experts in different cellular processes can speak the same modeling language.

At any extent, the *M. genitalium* organism remains a suitable model for whole-cell simulations, and its study has a medical interest as a consequence of its pathogenic nature. Therefore, the so obtained whole-cell biochemical network can provide useful information for the previously mentioned research fields, as can be further enhanced with the emergence of new data.

## Supplementary Information

Supplementary Information 1

Supplementary Information 2

## Data Availability

The scripts developed for parsing the WholeCellKB and genomic data into the *M. genitalium* whole-cell network, as well as the network files, are freely available in the Github repository https://github.com/pauloburke/whole-cell-network.
