# Supplementary material for: A biochemical network modeling of a whole-cell
Source: Sci Rep. 2020 Aug 6;10:13303. doi: 10.1038/s41598-020-70145-4 (PMC7411072; doi:10.1038/s41598-020-70145-4)
Supplement: Supplementary file 1 — Supplementary Information 1 [file 41598_2020_70145_MOESM1_ESM.pdf]

Supplementary Information
A Biochemical Network Modeling of a Whole-Cell

Paulo E. P. Burke, Claudia B. L. Campos, Luciano da F. Costa,
Marcos G. Quiles

1 The Modeling of Particular Interactions

1.1 Cell Division Reaction

The cell division is a biochemical and mechanical event involving several molecules and structures. In the *M. genitalium*'s whole-cell network it was modeled as a single reaction with all necessary molecules linked as modifiers. Figure S1 illustrates the reaction and Table S1.

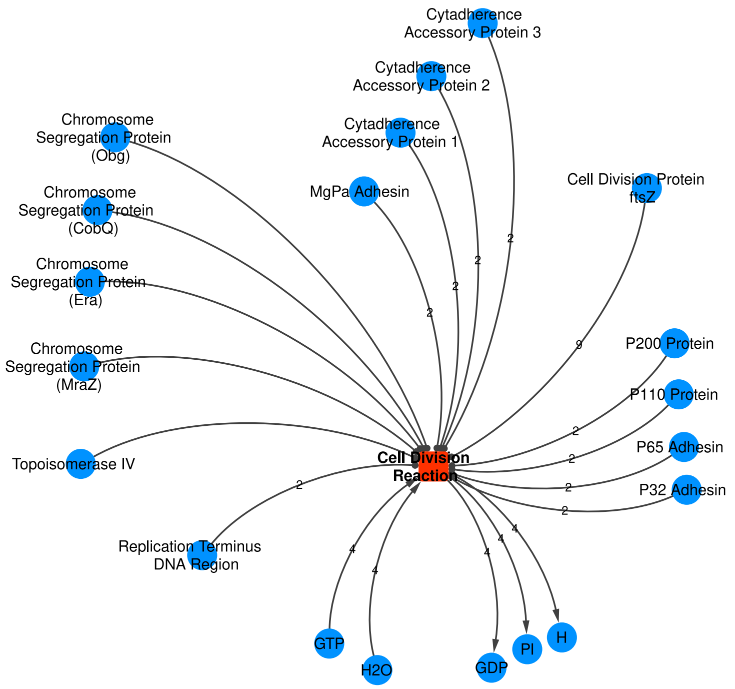

Figure S1: Cell Division Reaction illustration.

Table S1: Cell Division Reaction components.

Illustration Name	WholeCellKB WID	Network ID
**Reactions**		
Cell Division Reaction	-	cellular_division_reaction
**Molecules**		
GTP	GTP	c_GTP
GDP	GDP	c_GDP
H2O	H2O	c_H2O
PI	PI	c_PI
H	H	c_H
Replication Terminus DNA Region	-	c_Mgenitalium_Chr_1_region_2206_DNA
Chromosome Segregation Protein (MraZ)	MG_470_MONOMER	c_MG_470_MONOMER
Chromosome Segregation Protein (Era)	MG_384_MONOMER	c_MG_384_MONOMER
Chromosome Segregation Protein (CobQ)	MG_221_OCTAMER	c_MG_221_OCTAMER
Chromosome Segregation Protein (Obg)	MG_387_MONOMER	c_MG_387_MONOMER
Topoisomerase IV	MG_203_204_TETRAMER	c_MG_203_204_TETRAMER
Cell Division Protein ftsZ	MG_224_9MER_GDP	c_MG_224_9MER_GDP
MgPa Adhesin	MG_191_MONOMER	tm_MG_191_MONOMER
P110 Protein	MG_192_MONOMER	tm_MG_192_MONOMER
P200 Protein	MG_386_MONOMER	tc_MG_386_MONOMER
P32 Adhesin	MG_318_MONOMER	tm_MG_318_MONOMER
P65 Adhesin	MG_217_MONOMER	tc_MG_217_MONOMER
Cytadherence Accessory Protein 3	MG_317_MONOMER	tc_MG_317_MONOMER
Cytadherence Accessory Protein 2	MG_218_MONOMER	tc_MG_218_MONOMER
Cytadherence Accessory Protein 1	MG_312_MONOMER	tc_MG_312_MONOMER

1.2 Replication Reactions

The template for the replication reactions is described in the main document. Table S2 displays the information about the illustrated nodes.

Table S2: Replication components.

Illustration Name	WholeCellKB WID	Network ID
**Reactions**		
Replication Initiation	-	Mgenitalium_Chr_1_Replication_Initiation
Chromosome Region 0 Replication	-	Mgenitalium_Chr_1_region_0_DNA_Replication_Reaction
Chromosome Region 1 Replication	-	Mgenitalium_Chr_1_region_1_DNA_Replication_Reaction
Chromosome Region 2205 Replication	-	Mgenitalium_Chr_1_region_2205_DNA_Replication_Reaction
Chromosome Region 2207 Replication	-	Mgenitalium_Chr_1_region_2207_DNA_Replication_Reaction
Chromosome Region 4532 Replication	-	Mgenitalium_Chr_1_region_4532_DNA_Replication_Reaction
Chromosome Region 4533 Replication	-	Mgenitalium_Chr_1_region_4533_DNA_Replication_Reaction
Replication Terminus	-	Mgenitalium_Chr_1_region_2206_DNA_Replication_Reaction
**Molecules**		
dATP	dATP	c_dATP
dTTP	dTTP	c_dTTP
dCTP	dCTP	c_dCTP
dGTP	dGTP	c_dGTP
PPI	PPI	c_PPI
PI	PI	c_PI
Chromosome Region 0	-	c_Mgenitalium_Chr_1_region_0_DNA
Chromosome Region 1	-	c_Mgenitalium_Chr_1_region_1_DNA
Chromosome Region 2	-	c_Mgenitalium_Chr_1_region_2_DNA
Chromosome Region 4534	-	c_Mgenitalium_Chr_1_region_4534_DNA
Chromosome Region 4533	-	c_Mgenitalium_Chr_1_region_4533_DNA
Chromosome Region 4532	-	c_Mgenitalium_Chr_1_region_4532_DNA
Chromosome Region 2206	-	c_Mgenitalium_Chr_1_region_2206_DNA
DnaA ATP 7mer - DnaABox Region	-	c_Mgenitalium_Chr_1_region_4530_DNA_MG_469_7MER_ATP<br>c_Mgenitalium_Chr_1_region_4526_DNA_MG_469_7MER_ATP<br>c_Mgenitalium_Chr_1_region_4524_DNA_MG_469_7MER_ATP<br>c_Mgenitalium_Chr_1_region_4516_DNA_MG_469_7MER_ATP<br>c_Mgenitalium_Chr_1_region_4514_DNA_MG_469_7MER_ATP
DnaA ADP 7mer	MG_469_7MER_ADP	c_MG_469_7MER_ADP
DnaABox Regions	-	c_Mgenitalium_Chr_1_region_4530_DNA<br>c_Mgenitalium_Chr_1_region_4526_DNA<br>c_Mgenitalium_Chr_1_region_4524_DNA<br>c_Mgenitalium_Chr_1_region_4516_DNA<br>c_Mgenitalium_Chr_1_region_4514_DNA
Replication Complex Region 0	-	c_Mgenitalium_Chr_1_region_0_Replication_Complex
Replication Complex Region 1	-	c_Mgenitalium_Chr_1_region_1_Replicating_Complex
Replication Complex Region 4534	-	c_Mgenitalium_Chr_1_region_4534_Replication_Complex
Replication Complex Region 4533	-	c_Mgenitalium_Chr_1_region_4533_Replicating_Complex
Replication Complex Region 2206	-	c_Mgenitalium_Chr_1_region_2206_Replicating_Complex
DNA Topoisomerase I	MG_122_MONOMER	c_MG_122_MONOMER
DNA Topoisomerase IV	MG_203_204_TETRAMER	c_MG_203_204_TETRAMER
DNA Gyrase	DNA_GYRASE	c_DNA_GYRASE
DNA Primase	MG_250_MONOMER	c_MG_250_MONOMER
DNA Helicase	MG_094_HEXAMER	c_MG_094_HEXAMER
DNA Polymerase III Beta	MG_001_MONOMER	c_MG_001_MONOMER
DNA Polymerase Gamma Complex	DNA_POLYMERASE_GAMMA_COMPLEX	c_DNA_POLYMERASE_GAMMA_COMPLEX
DNA Polymerase Core	DNA_POLYMERASE_CORE	c_DNA_POLYMERASE_CORE

1.3 Transcription Reactions

The template for the transcription reactions is described in the main document. Table S3 displays the information about the illustrated nodes. Once these re-

actions are templates, the exact name of molecules and reactions depends on the gene in the subject. Thus, we use the placeholder *GENE* which can stand for the gene's name or transcription units for single and polycistronic genes respectively. The placeholder *CHRM_REG* stands for the chromosome region.

Table S3: Transcription components.

**Illustration Name**	**WholeCellKB WID**	**Network ID**
**Reactions**		
Transcription Complex Formation	-	*GENE*_Transcription_Complex_Formation
Transcription Elongation	-	*CHRM_REG*_*GENE*_Transcription_Elongation
Transcription End	-	*GENE*_Transcription_End
RNA Cleavage	-	*GENE*_Processing
Maturation Reaction	-	*GENE*_Maturation
**Molecules**		
ATP	ATP	c_ATP
UTP	UTP	c_UTP
CTP	CTP	c_CTP
GTP	GTP	c_GTP
PPI	PPI	c_PPI
RNA Polymerase	RNA_POLYMERASE	c_RNA_POLYMERASE
RNA Polymerase Holoenzyme	RNA_POLYMERASE_HOLOENZYME	c_RNA_POLYMERASE_HOLOENZYME
Sigma Factor	MG_249_MONOMER	c_MG_249_MONOMER
Transcription Factor	-	Depends on the *GENE*
DNA Region i with Transcription Factor	-	Depends on the *GENE*
Transcription Complex	-	c_Mgenitalium_Chr_1_region_i_DNA_*GENE*_Transcription_Complex
Transcribing Complex	-	c_Mgenitalium_Chr_1_region_i+1_DNA_*GENE*_Transcribing_Complex
DNA Region i	-	c_Mgenitalium_Chr_1_region_i_DNA
DNA Region i+1	-	c_Mgenitalium_Chr_1_region_i+1_DNA
Transcription Elongation Factors	MG_282_MONOMER	c_MG_282_MONOMER
Transcription Release Factors	MG_027_MONOMER<br>MG_141_MONOMER	c_MG_027_MONOMER<br>c_MG_141_MONOMER
RNA		c_*GENE*
RNAse	MG_0003_465<br>MG_110_MONOMER<br>MG_139_DIMER<br>MG_367_DIMER<br>MG_425_DIMER	c_MG_0003_465<br>c_MG_110_MONOMER<br>c_MG_139_DIMER<br>c_MG_367_DIMER<br>c_MG_425_DIMER
Imature RNA	-	c_Imature_*GENE*
Modification Metabolites	-	Depends on the *GENE*
Modification Enzymes	-	Depends on the *GENE*
Modification Side Products	-	Depends on the *GENE*

1.4 Transcription Stall Reactions

A transcription reaction can be interrupted for several reasons. One of them is the collision with other molecules in the same region of a DNA strand. Here we modeled the stall reaction for transcribing complexes when a replication complex is in the next chromosome region. Once the transcription reaction can be interrupted at many chromosome regions, one incomplete RNA molecule is created for each reaction. The name of the molecule carries its sequence.

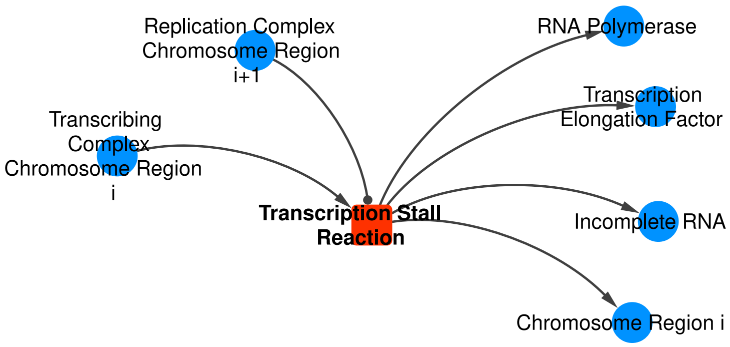

Figure S2: Transcription Stall Template.

Table S4: Transcription Stall components.

Illustration Name	WholeCellKB WID	Network ID
**Reactions**		
Transcription Stall Reaction	-	*GENE*_Transcription_Complex_Stall
**Molecules**		
Transcribing Complex Chromosome Region i	-	c_Mgenitalium_Chr_1_region_i+1_DNA_*GENE*_Transcribing_Complex
Replication Complex Region i+1	-	c_Mgenitalium_Chr_1_region_i+1_Replication_Complex
DNA Region i	-	c_Mgenitalium_Chr_1_region_i_DNA
RNA Polymerase	RNA_POLYMERASE	c_RNA_POLYMERASE
Transcription Elongation Factors	MG_282_MONOMER	c_MG_282_MONOMER
Incomplete RNA		c_RNA_*SEQUENCE*

1.5 RNA Degradation Reactions

The RNA degradation reaction template is depicted in Figure S3. The Peptidyl-tRNA Hydrolase is needed only in the case of aminoacylated tRNAs. Modifications in RNAs were not taken into account due to inconsistencies in WholeCellKB. Table S5 shows the component's names in WholeCellKB and the network model.

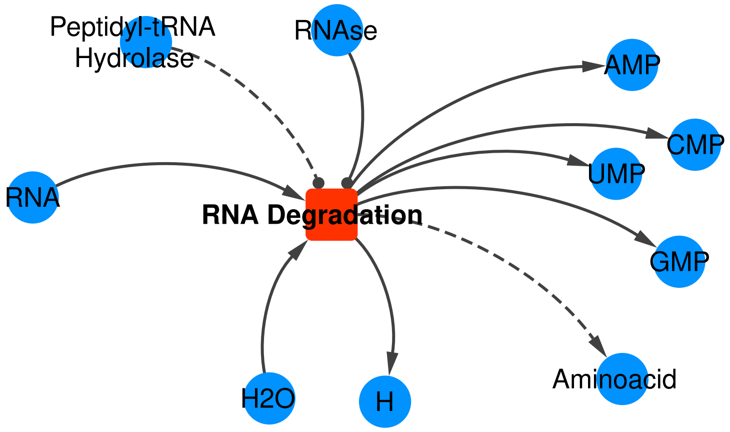

Figure S3: RNA Degradation Template.

Table S5: RNA Degradation components.

Illustration Name	WholeCellKB WID	Network ID
**Reactions**		
RNA Degradation	-	*RNA*_Degradation
**Molecules**		
ATP	ATP	c_AMP
UMP	UMP	c_UMP
CMP	CMP	c_CMP
GMP	GMP	c_GMP
PPI	PPI	c_PPI
H2O	H2O	c_H2O
H	H	c_H
RNAse	MG_104_MONOMER	c_MG_104_MONOMER
Peptidyl-tRNA Hydrolase	MG_083_MONOMER	c_MG_083_MONOMER
RNA	-	Depends on the *RNA*
Aminoacid	-	Depends on the *RNA*

1.6 Translation Reactions

The template for the translation reactions is described in the main document. Table S6 displays the information about the illustrated nodes. Once these reactions are templates, the exact name of molecules and reactions depends on the gene in the subject. Thus, we use the placeholder *GENE* which can stand for the gene's name. The placeholder *LOC* stands for location, which can be: cytosol (c), membrane (m), extracellular (e). The placeholder *PROT* is indi-

cated in the table as the Protein Monomer. Table S7 lists all the amino acids in the model. Table S8 shows all the tRNAs and their respective amino acid and codons.

Table S6: Translation components.

Illustration Name	WholeCellKB WID	Network ID
**Reactions**		
Translation Complex Formation	-	*PROT*_Translation_Complex_Formation
Translation Elongation	-	*PROT*_Translation_Elongation
Maturation Reaction	-	*PROT*_Maturation
**Molecules**		
GTP	GTP	c_GTP
GDP	GDP	c_GDP
H2O	H2O	c_H2O
PI	PI	c_PI
H	H	c_H
Ribosome 70S	RIBOSOME_70S	c_RIBOSOME_70S
IF-1	MG_173_MONOMER	c_MG_173_MONOMER
IF-2	MG_142_MONOMER	c_MG_142_MONOMER
IF-3	MG_196_MONOMER	c_MG_196_MONOMER
	MG_026_MONOMER	c_MG_026_MONOMER
	MG_089_DIMER	c_MG_089_DIMER
Elongation Auxiliaries	MG_258_MONOMER	c_MG_258_MONOMER
	MG_433_DIMER	c_MG_433_DIMER
	MG_435_DIMER	c_MG_435_DIMER
	MG_451_DIMER	c_MG_451_DIMER
mRNA	-	Depends on the *PROT*
Chaperones	-	Depends on the *PROT*
Translation Complex	-	c_*PROT*_Translation_Complex
Imature Protein	-	c_Imature_*PROT*
Protein Monomer	-	*LOC_GENE*_MONOMER (*PROT*)
Modification Metabolites	-	Depends on the *PROT*
Modification Enzymes	-	Depends on the *PROT*
Modification Side-Products	-	Depends on the *PROT*
Membrane Transporters	-	Depends on the *PROT*

Table S7: Aminoacids

Name	WholeCellKB WID	Network ID
Alanine	ALA	c_ALA
Arginine	ARG	c_ARG
Asoaragine	ASN	c_ASN
Aspartic Acid	ASP	c_ASP
Cysteine	CYS	c_CYS
Formyl-Methionine	FMET	c_FMET
Glutamine	GLN	c_GLN
Glutamic Acid	GLU	c_GLU
Glycine	GLY	c_GLY
Histidine	HIS	c_HIS
Isoleucine	ILE	c_ILE
Leucine	LEU	c_LEU
Lysine	LYS	c_LYS
Methionine	MET	c_MET
Phenylalanine	PHE	c_PHE
Proline	PRO	c_PRO
Serine	SER	c_SER
Threonine	THR	c_THR
Tryptophan	TRP	c_TRP
Tyrosine	TYR	c_TYR
Valine	VAL	c_VAL

Table S8: tRNAs

tRNA WCKB WID	Aminoacid	tRNA Network ID	Aminoacylated tRNA Network ID
MG471	ALA	c_MG471	c_MG471_ALA
MG472	ILE	c_MG472	c_MG472_ILE
MG475	SER	c_MG475	c_MG475_SER
MG479	THR	c_MG479	c_MG479_THR
MG483	CYS	c_MG483	c_MG483_CYS
MG484	PRO	c_MG484	c_MG484_PRO
MG485	MET	c_MG485	c_MG485_MET
MG486	ILE	c_MG486	c_MG486_ILE
MG487	SER	c_MG487	c_MG487_SER
MG488	FMET	c_MG488	c_MG488_FMET
MG489	ASP	c_MG489	c_MG489_ASP
MG490	PHE	c_MG490	c_MG490_PHE
MG492	ARG	c_MG492	c_MG492_ARG
MG493	GLY	c_MG493	c_MG493_GLY
MG495	ARG	c_MG495	c_MG495_ARG
MG496	TRP	c_MG496	c_MG496_TRP
MG497	ARG	c_MG497	c_MG497_ARG
MG499	GLY	c_MG499	c_MG499_GLY
MG500	LEU	c_MG500	c_MG500_LEU
MG501	LYS	c_MG501	c_MG501_LYS
MG502	GLN	c_MG502	c_MG502_GLN
MG503	TYR	c_MG503	c_MG503_TYR
MG504	TRP	c_MG504	c_MG504_TRP
MG506	SER	c_MG506	c_MG506_SER
MG507	SER	c_MG507	c_MG507_SER
MG508	LEU	c_MG508	c_MG508_LEU
MG509	LYS	c_MG509	c_MG509_LYS
MG510	THR	c_MG510	c_MG510_THR
MG511	VAL	c_MG511	c_MG511_VAL
MG512	THR	c_MG512	c_MG512_THR
MG513	GLU	c_MG513	c_MG513_GLU
MG514	ASN	c_MG514	c_MG514_ASN
MG518	HIS	c_MG518	c_MG518_HIS
MG519	LEU	c_MG519	c_MG519_LEU
MG520	LEU	c_MG520	c_MG520_LEU
MG523	ARG	c_MG523	c_MG523_ARG

1.7 Translation Stall Reactions

Just as transcription reactions, the translation process can be interrupted by several reasons too. However, when a transcription complex stalls, the incomplete protein needs to be tagged with a specific amino acid sequence in order to be rapidly degraded. Thus, this process is represented by two template reactions: the stall of the translation complex and the translation of the signal peptide. Once we do note represent intermediate molecules during the translation process, all stalled translation reactions will only produce the same incomplete peptide, which contains only the degradation signal sequence. The reactions' templates are described in Figure S4 and Table S9.

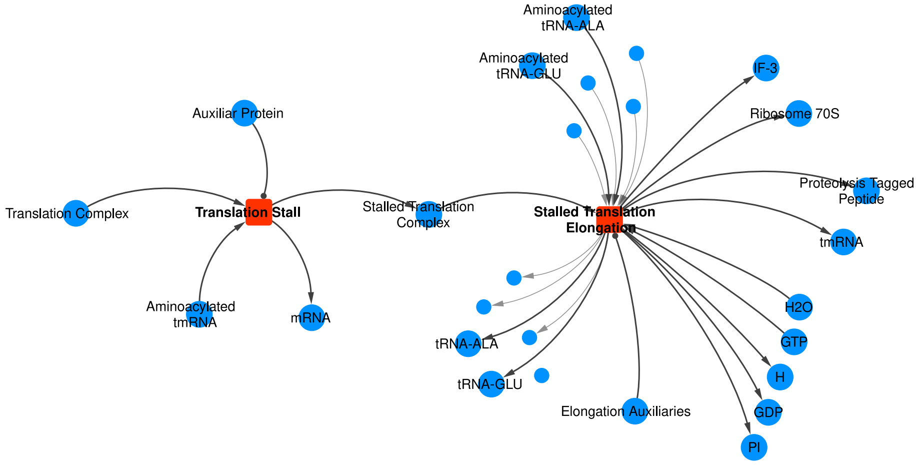

Figure S4: Translation Stall Template.

Table S9: Translation Stall components.

Illustration Name	WholeCellKB WID	Network ID
**Reactions**		
Translation Stall	-	*PROT*_Translation_Complex_Stall
Stalled Translation Elongation	-	Stalled_*PROT*_Translation_Complex_Translation_Elongation
**Molecules**		
GTP	GTP	c_GTP
GDP	GDP	c_GDP
H2O	H2O	c_H2O
PI	PI	c_PI
H	H	c_H
Ribosome 70S	RIBOSOME_70S	c_RIBOSOME_70S
IF-3	MG_196_MONOMER	c_MG_196_MONOMER
Elongation Auxiliaries	MG_026_MONOMER	c_MG_026_MONOMER
	MG_089_DIMER	c_MG_089_DIMER
	MG_258_MONOMER	c_MG_258_MONOMER
	MG_433_DIMER	c_MG_433_DIMER
	MG_435_DIMER	c_MG_435_DIMER
	MG_451_DIMER	c_MG_451_DIMER
mRNA	-	Depends on the *PROT*
Translation Complex	-	c_*PROT*_Translation_Complex
Stalled Translation Complex	-	c_Stalled_*PROT*_Translation_Complex
tmRNA	MG_0004	c_MG_0004
Aminoacylated tmRNA	-	c_MG_0004_ALA
Proteolysis Tagged Peptide	-	c_Peptide_ACKSKVNTCLLVNDQIWIYQHFVIFFV

1.8 Protein Degradation Reactions

The proteins produced by the cell can be degraded in order to recycle amino acids, control proteins' concentration, remove defective proteins from the cytosol, and other reasons. Figure S5 and Table S10 shows the template for protein degradation reactions. According to the protein's location (cytosol or membrane), different proteases can be recruited for its degradation. Proteins tagged with the Proteolysis Peptide are degraded by the membrane protease.

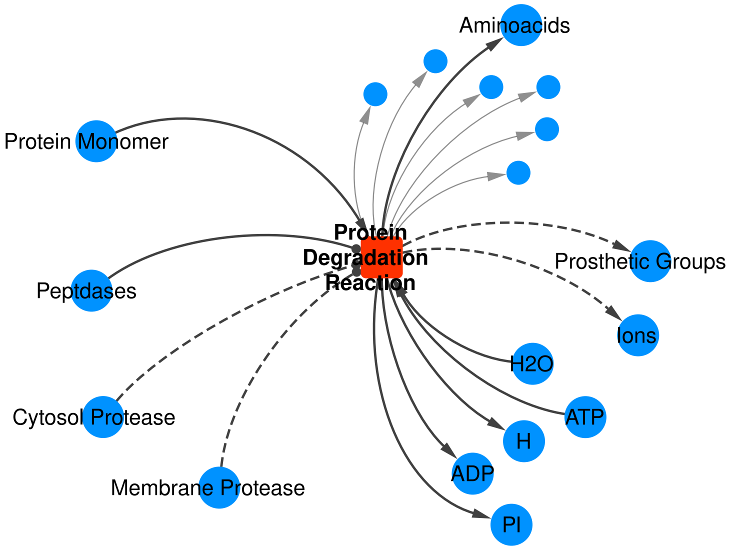

Figure S5: Protein Degradation Template.

Table S10: Protein Degradation components.

Illustration Name	WholeCellKB WID	Network ID
**Reactions**		
Protein Degradation Reaction	-	*PROT*_Degradation
**Molecules**		
ATP	ATP	c_ATP
ADP	ADP	c_ADP
H2O	H2O	c_H2O
PI	PI	c_PI
H	H	c_H
Protein Monomer	-	Depends on the *PROT*
Aminoacids	-	Depends on the *PROT* composition
Prosthetic Groups	-	Depends on the *PROT* composition
Ions	-	Depends on the *PROT* composition
Cytosol Protease	MG_239_HEXAMER	c_MG_239_HEXAMER
Membrane Protease	MG_457_HEXAMER	m_MG_457_HEXAMER
Peptdases	MG_020_MONOMER	c_MG_020_MONOMER
	MG_046_DIMER	c_MG_046_DIMER
	MG_183_MONOMER	c_MG_183_MONOMER
	MG_208_DIMER	c_MG_208_DIMER
	MG_324_MONOMER	c_MG_324_MONOMER
	MG_391_HEXAMER	c_MG_391_HEXAMER

2 Software Structure and Implementation

The software called PiCell was developed to build the Whole-Cell Extended Biochemical Network of *Mycoplasma genitalium* but also being adaptable for other organisms. It is composed of three parts:

- Database Handler
- PiCell Core
- Network Constructor

that can be accessed by Python 3 scripts. The database handler is the interface between databases and the PiCell core. One handler should be implemented for each database to be used as a source of the model. The PiCell Core is responsible for organizing the data obtained from databases and create intermediate molecules and reactions in order to fulfill the central dogma of biology in the model. When all necessary information os gathered in the PiCell Core, it can be exported as a single network model, with linked molecule and reaction nodes, following the framework proposed in this work. This model is then

further submitted to validation and analyses. In Figure S6 the reader can find a schematic of the software implemented to build the *M. genitalium*'s network.

2.1 Database Handler

The necessary information for the model was acquired from the WholeCellKB through the WholeCellKB Handler, a piece of Python 3 code implemented specifically for this database. The data in the WholeCellKB database was available in several formats. The JSON format was chosen because of its easiness of access from Python. In addition to the JSON database file, the Handler can read two other files: one containing the database entries to be ignored, and another containing a name mapping to be applied in the database.

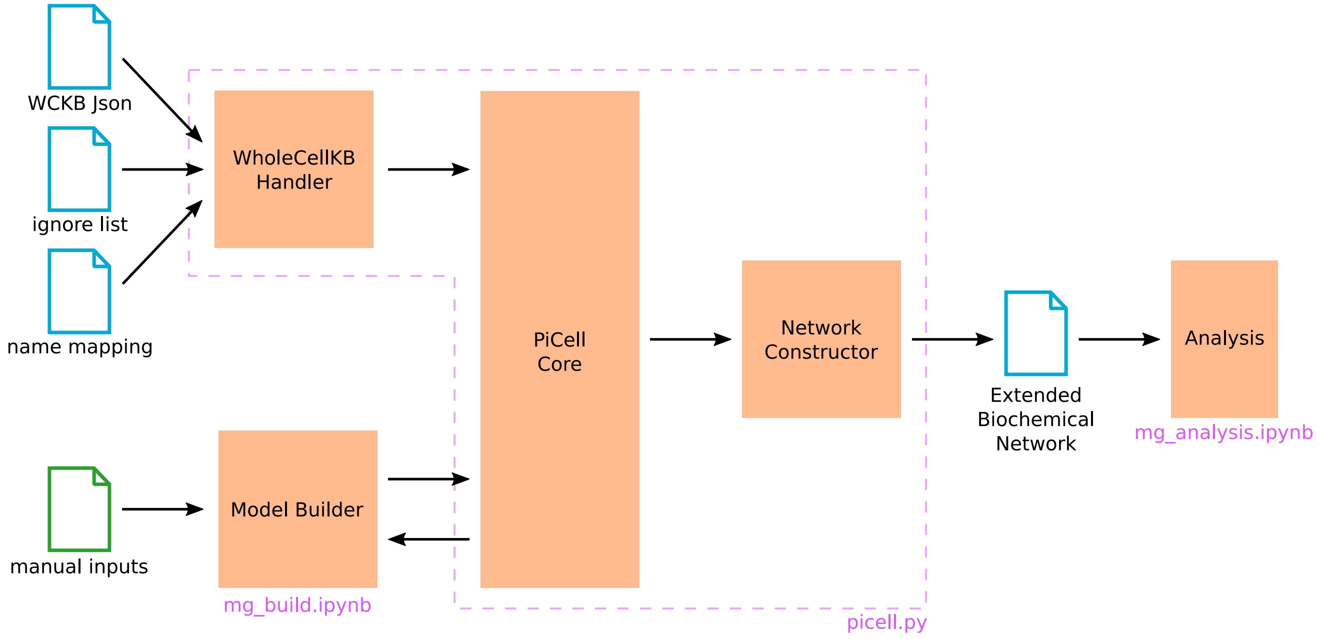

Figure S6: The schematic implementation of the PiCell, a software to build Whole-Cell Extended Biochemical Networks.

2.2 Model Builder

The control of the modeling is made through an IPython Notebook using the Jupyter interface. Before acquiring the database's information, the model must be configured. Information about the canonical cellular processes must be provided in order to be constructed from the templates by the PiCell Core. The

information provided is described in the Tables S2 to S10.

The genetic information about the organism must also be provided. In the case of *M. genitalium*, it was also available in the WholeCellKB. The chromosome sequence, chromosome features, genes, and transcription units are necessary to construct the canonical processes.

Molecules and reactions to be added in the model can be retrieved from the database or inserted manually. An example of the latter is the cell division reaction and its structure and components can be found in Figure S1 and Table S1. Reactions, such as metabolic and aminoacylation, were retrieved from the database, as well as the participant molecules.

2.3 PiCell Core

The PiCell Core is responsible to structure the information acquired from databases and inserted manually in such a way that it can be more easily manipulated, checked for inconsistencies, and be further translated into an extended biochemical network.

Chromosome Representation The first function of the PiCell Core is to create a representation of the cell's chromosomes based on the genetic information provided. Each chromosome is divided into regions according to annotated regions and respecting a maximum region length. In the case of *M. genitalium*, the maximum region length was set a very high value so that all the regions' sizes are only constrained by the annotations in the genome. Transcription Units' starts and ends were not considered in this process.

Recursive Creation of Canonical Reactions The second function of the PiCell Core is to generate missing canonical reactions for macromolecules in the model. This functionality is based on the premise that all macromolecules in the model must have at least one biosynthesis and one degradation reaction. Thus, this process can iterate from protein complexes needing their complexation reaction, up to the expression of their respective genes. For example, consider that a given metabolic reaction inserted in the model is catalyzed by a protein complex. The complex must be synthesized by a protein complexation reaction. The monomers required in this reaction must be synthesized by a translation reaction from their respective mRNA. The mRNA then needs to be synthesized by a transcription reaction from its respective DNA regions. Finally, DNA regions must be synthesized by their replication reactions. This cycle of reactions must be created for every macromolecule in the model. Similarly, the degradation reactions for each macromolecule is created. All reactions created by the PiCell Core are based on the templates described before. Particularities of each reaction created, such as specific chaperones in protein translation, are added in the reactions according to data availability in the database.

Consistency Checks Additionally to the premise presented in the last paragraph, the PiCell Core performs a mass-balance check in order to probe for inconsistencies in the reactions. All metabolites must have their composition formula described in the model. From their atomic composition, their mass is estimated. Given that all macromolecules are combinations of basic metabolites, the mass of all molecules can be estimated upwards. Then, to check the mass-balance consistency of any reaction, we simply calculate the mass of reactants minus the mass of products. The absolute value obtained must be less than one, the mass of a hydrogen atom. It is important to notice that although this methodology adds an extra layer of confidence in the model, the correctness of all reactions still relies on the data sources.

Extended Biochemical Network Construction After the model completion, it is ready to generate a working model following the extended biochemical network framework. For each reaction described in the PiCell core, a respective reaction is created in the network. The molecules are created respecting their location. If a given molecule can occur in more than one location, one molecule node is created for each location and linked to their respective reactions accordingly. Reversible reactions are represented by two reaction nodes, one for each direction. The final model can be exported in SBML, some network formats, and also as a networkx graph. The data formats are described in Section 3.

2.4 Software Dependencies

The PiCell is developed using Python 3 and it depends on some Python Packages. The packages are all open source and are listed in the following:

- json
- molmass
- networkx
- libsbml

For the scripts used in the analysis of the model, you will also need the following packages:

- numpy
- matplotlib
- openpyxl
- scipy

3 Network's Data Formats

The *M. genitalium*'s whole-cell biochemical network is available in three formats, SBML, GML, and GraphML (Additional file 2).

mg_network.sbml Molecule nodes are stored as Species objects and reaction nodes as Reaction objects. The common reaction representation of SBML models is used but without kinetic laws associated. Catalytic molecules, such as enzymes, are connected to reactions as modifiers. The SBML file does not contain annotations for molecules or reactions. Also, SBML does not support setting the stoichiometry for the modifiers in reactions. Thus, we always recommend to use the GML or GraphML formats.

mg_network.gml and .graphml All the main nodes and edges attributes are described below. Particular nodes may contain more attributes with additional information.

General node attributes:

- annotations: Annotations from WholeCellKB and the authors
- crossreferences: From WholeCellKB
- degree: Number of connections
- indegree: number of inward connections
- instodegree: number of inward connections weighted by their stoichiometry
- name: Unique ID for each node
- originaldatabase: Whether it is from WholeCellKB or created by PiCell
- outdegree: Number of outward connections
- outstodegree: Number of outward connections weighted by their stoichiometry
- stodegree: Number connections weighted by their stoichiometry
- type: Molecule (m) or reaction (r)
- usualname: Human readable name

Only molecule nodes' attributes:

- compartment: cytosol (c), membrane (m), extracellular (e), terminal organelle cytosol (tc), terminal organelle membrane (tm)
- moltype: Type of molecule (metabolite, protein, RNA, DNA, etc.)
- mw: Molecular weight

Only reaction nodes' attributes:

- isreversible: If the reaction is reversible (True/False)

- process: Process where the reaction occurs
- reacttype: Type of the reaction (Metabolic, Protein Synthesis, etc.)

Edge attributes:

- type: type of connection: reactant (r), product (p), modifier (m)
- sto: stoichiometry

Cascading failure algorithm

Algorithm 1 Cascading Failure

```
procedure recursiveNodeRemoval(N, TARGET_REACTION)    ▷ N is the molecule node to remove
    R ← list of reactions where N is reactant
    M ← empty list of molecules
    remove(N)
    while Length(R) > 0 do
        for all r in R do
            for all products of r do
                if product.indegree = 0 then
                    append product to M
            remove(r)
        R ← empty list of reactions
        for all m in M do
            append reactions where m is reactant to R
            remove(m)
        M ← empty list of molecules
```
